# Effect of maternal polycystic ovary syndrome (PCOS) on screening of aneuploidy in the first and second trimesters

**DOI:** 10.1186/s13048-023-01251-w

**Published:** 2023-08-21

**Authors:** Narjes Hassan Haivadi, Shahideh Jahanian Sadatmahalleh, Fatemeh Razavinia, Sarang Younesi, Malihe Nasiri, Saeideh Ziaei

**Affiliations:** 1 Department of Reproductive Health and Midwifery, Faculty of Medical Sciences, Tarbiat Modares University, Tehran, Iran; 2 Department of Midwifery, School of Nursing & Midwifery, Ahvaz Jundishapur University of Medical Sciences, Ahvaz, Iran; 3 Nilou Medical Laboratory, Tehran, Iran; 4 Department of Basic Sciences, Faculty of Nursing and Midwifery, Shahid Beheshti University of Medical Sciences, Tehran, Iran

**Keywords:** Polycystic ovary syndrome, First-trimester screening test, Second-trimester screening test, Aneuploidy

## Abstract

**Background:**

Polycystic ovary syndrome (PCOS) is characterized by insulin resistance and hormonal disorder in women. This study aimed to assess the effect of maternal PCOS on screening of aneuploidy in the first and second-trimesters.

**Methods:**

This case-control study was conducted in Arash Hospital and Nilou Laboratory in 2017–2018. The screening test was conducted on 90 PCOS and 90 healthy mothers. Finally, the first and second-trimester screening was compared between the two groups using Chi-square, Mann-Whitney’s U and students T tests and regression model by SPSS 21. P < 0.05 was considered as statistically significant.

**Results:**

Free Beta-Human Chorionic Gonadotropin (Free-β-HCG) (P = 0.04), inhibin-A (P = 0.001) and Alpha Fetoprotein (AFP) (P = 0.02) levels were higher in the PCOS women comparing to the healthy women but there was no significant difference between the mean of HCG, Plasma Protein A (PAPP-A), and Unconjugated Estriol (UE3) between the two groups. Pre-eclampsia (P < 0.001) and trisomy 18 risks in quad screening were higher in the PCOS women (P = 0.002**)** than the control group; however, trisomy 13, trisomy 18 and trisomy 21 risks, Smith-Lemli-Opitz Syndrome (SLOS) and Neural Tube Defect (NTD) risks were not different between the two groups. The logistic regression model showed that the first- and second-trimester screening of aneuploidywas related to PCOS.

**Conclusions:**

There was a significant difference in the mean of free**-**β-HCG, inhibin-A, AFP level, and the risks of pre-eclampsia, SLOS and trisomy 18 between the two groups but no significant association was found in the mean of HCG, PAPP-A, UE3, NTD and other aneuploidies between the two groups. PCOS may affect the first- and second-trimester screening tests and pregnancy health. It may also require correction in the calculation of risks related to the first- and second-trimester screening for aneuploidy.

## Background

Polycystic ovary syndrome (PCOS), as one of the most common maternal endocrine disorders worldwide (6–10%) [1], is characterized by hyperandrogenism, anovulation, and polycystic ovary. It is also associated with insulin resistance, diabetes, obesity and hormonal disorder [2]. Insulin resistance and hyper-insulinemia have been implicated in steroidogenic dysfunction of the ovary [3]. In PCOS women, LH (Luteinizing Hormone) level is higher than FSH (Follicle Stimulating Hormone). Increasing the level of LH may increase testosterone secretion in Theca cells and decrease inhibin secretion in granulosa cells in the ovary [4]. The similar structure of LH and HCG (Human Chorionic Gonadotropin) can increase the HCG level in pregnant women with PCOS [5].

During pregnancy, the placenta secrets biochemical markers such as plasma protein A (PAPP-A), free beta-human chorionic gonadotropin (free β-hCG), HCG, AFP (Alpha Fetoprotein), UE3 (Unconjugated Estriol), and inhibin-A. The amount of these biochemical markers changes in aneuploidies and high-risk pregnancy [6–11]. On the other hand, PAPP-A, free β-hCG and nuchal translucency (NT) in the first-trimester and UE3, inhibin-A, HCG and AFP in the second trimester predict aneuploidy, Neural Tube Defect (NTD) and high-risk pregnancy [12]. As a result, a hormonal disorder in women with PCOS may modify the screening of biochemical markers in the first and second trimesters of pregnancy [13]. An abnormal screening result increases stress, invasive assessment, unnecessary abortion, and economic burden during pregnancy. Hence, due to the importance of the issue, the present study was conducted to assess the effect of maternal PCOS on the first trimester (integrated :NT, PAPP-A, and free-β-hCG) and second trimester (quad marker :HCG, AFP, UE3, and inhibin-A) screening of aneuploidy.

## Methods

This case-control study was conducted among 180 first- and second-trimester pregnant (90 PCOS and 90 healthy control) women meeting our eligibility criteria and referring to Arash Hospital and Nilou Laboratory in Tehran/Iran between October 2017 and March 2018. The study protocol was approved by the Ethics Committee of Tarbiat Modares University, Tehran/Iran (IR.TMU.REC.139.5369).

The sample size for this study was considered based on the study of Hacivelioglu et al. [13] with the 95% confidence interval (CI) and 80% test power, 83 subjects were considered in each group and with withdrawal rate (about 10%) 90 subjects were considered in each group and a total of 180 subjects.

The eligibility criteria for participation were: women aged 18–35 years, gestational age 11 to 18 week and 6 days, singleton pregnancy, no history of chronic diseases (such as diabetes mellitus, hypertension, thyroid and criteria), absence of any chromosomal abnormality in the fetus, no smoking, and no alcohol drinking.

The exclusion criteria were as follows: pregnancy undergoing with Assisted Reproductive Techniques (ART), In Vitro Fertility (IVF) or Intra Cytoplasmic Sperm Injection (ICSI), complicated pregnancy, Cushing’s syndrome, adrenal hyperplasia, and adrenal tumor.

Oligomenorrhea or amenorrhea, hyperandrogenism, hirsutism and number of follicles > 12 in sonography (Rotterdam criteria) were selected as the PCOS group [14]. Healthy women with regular menstrual cycles (21–35 days) were identified as the control group. Gestational age (GA) was calculated by ultrasonic examination in < 12 weeks and the demographic test was obtained through direct interview.

Fetus NT, crown-rump length (CRL) and any major fetal abnormalities were evaluated by ultrasound. Maternal serum PAPP-A and free-β-HCG were measured using the Krypton analyzer in 11 to 13 weeks and 6 day. UE3, inhibin-A, and AFP were evaluated using ELISA, and HCG was measured using Electro Chemi Luminescence (ECC) in 15 to 18 weeks and 6 day. The sensitivity of the first trimester screening test was 80%, its spasticity was 90%, and false positive was 5%. The results of the first trimester screening test were as fallowes: less than 100 multiples of median (MoM) ( high-risk), 100 to 1000 MoM ( borderline), and more than 1,000 MoM (low-risk). The sensitivity of the second trimester screening test was 78–81%, and the results of the second trimester screening test were as fallowes: less than 250 MoM (high-risk), 250 to 1000 MoM (borderline), and more than 1,000 MoM (low-risk) [15].

Finally, the first- and second-trimester screening tests results were compared between the two groups, and the risks of aneuploidy, NTD, Smith-Lemli-Opitz Syndrome (SLOS) and pre-eclampsia were assessed.

### Statistical analysis

Statistical analysis was performed using the SPSS software (ver. 21) for windows (SPSS, Inc., Chicago, IL, USA), and data are given as mean ± SD and number (%). Non-parametric Mann-Whitney’s U-test, students T-test, and Chi-square test were used to compare the categorical variables. Logistic regression was employed to examine associations between the outcomes such as the first- and second-trimester screening markers in the two groups and to estimate their odds ratio with 95% confidence interval. P < 0.05 was considered significant.

## Results

Table 1 indicates the demographic characteristic of the participating mothers. As shown, there is no significant difference in demographic characteristics between the two groups. Table 2 summarizes the screening test results in the first and second trimesters of the study population. As shown, there is a significant difference between the mean of free-β-HCG between two groups (P = 0.04). Also the mean of AFP level in the PCOS group is significantly higher than in the control group (38.34 ± 15.91 vs. 32.14 ± 15.13, P = 0.02). The mean of inhibin-A is 276.14 ± 129.38 in the PCOS group and 223.44 ± 102.07 in the control group, showing a significant association between the two groups in this regard (P = 0.001); however, no significant difference was observed in PAPP-A, HCG and UE3 levels between the PCOS and control groups.

**Table 1:** Homogeneity of the two groups of PCOS mothers and controls in terms of demographic characteristics

Variables	PCOS (n = 90)	Control (n = 90)	P-value
Age ( year)*	27.68 ± 4.51	27.51 ± 4.40	0.64
GA (week)** (in the first trimester)	12.40 ± 0.65	12.50 ± 0.64	0.98
GA (week)** (in the second trimester)	17 ± 1.30	16.6 ± 1.20	0.42
CRL (cm)*	12.40 ± 0.72	12.50 ± 0.67	0.08
Education***			0.12
Secondary school degree (< 8 years)	9 (10.10)	14 (15.60)	
Diploma (< 12 years)	46 (51.10)	48 (59.30)	
High school diploma (13 years)	10 (11.10)	11 (12.20)	
Bachelor (15 years)	21 (23.30)	15 (16.70)	
Master of science (17 years)	4 (4.40)	2 (2.20)	
Work***			0.50
Housewives	80 (88.9)	81 (90)	
Employee	10 (11.10)	9 (10)	
Gravid***			0.50
1	62 (68.90)	53 (58.90)	
2	21 (22.30)	35 (38.90)	
3	7 (77.80)	3 (2.20)	
BMI (kg/m^2^)*	25.51 ± 4.40	24.65 ± 3.81	
≤ 19.8	7 (3.9)	9 (5)	0.72
19.8–24	3 (17.8)	27 (15)	
24–26	10 (5.6)	24 (13.30)	
26–30	30 (16.70)	23 (12.80)	
≥ 30	11 (6.10(	7 (3.90)	

PCOS: Polycystic Ovary Syndrome, GA: Gestational Age, CRL: Crown-Rump Length, BMI: Body Mass Index

*Values are given as mean ± SD using Mann-Whitney’s U test

**Values are given as mean ± SD using students T-test

*** Values are given as a number (%) using Chi-squared test

**Table 2:** Comparison of screening markers in the PCOS and control groups

Variables	PCOS (n = 90)	Control (n = 90)	P-value
NT (mm)*	1.6 ± 0.38	1.41 ± 0.36	0.37
Free β-HCG (ng/ml)*	34.42 ± 18.92	28.99 ± 18.80	**0.04**
PAPP-A (mu/ml)*	4903.80 ± 8571.87	2994.93 ± 1626.76	0.59
AFP (ng/ml)**	38.34 ± 15.91	32.14 ± 15.13	**0.02**
HCG (mu/ml)*	19496.31 ± 16694.98	177199.21 ± 18877.56	0.11
UE3 (ng/ml)*	2.3 ± 1.89	2.01 ± 1.12	0.45
Inhibin-A (pg/ml)*	276.14 ± 129.38	223.44 ± 102.07	**0.001**

PCOS: Polycystic Ovary Syndrome, NT: Nuchal Translucency, Free β-HCG: Free Beta-Human Chorionic Gonadotropin, PAPP-A: Plasma Protein A, AFP: Alpha Fetoprotein, HCG: Human Chorionic Gonadotropin, UE3: Unconjugated Estriol

*Values are given as mean ± SD using Mann-Whitney’s U test

**Values are given as mean ± SD using students T-test

**Fig. 1:**
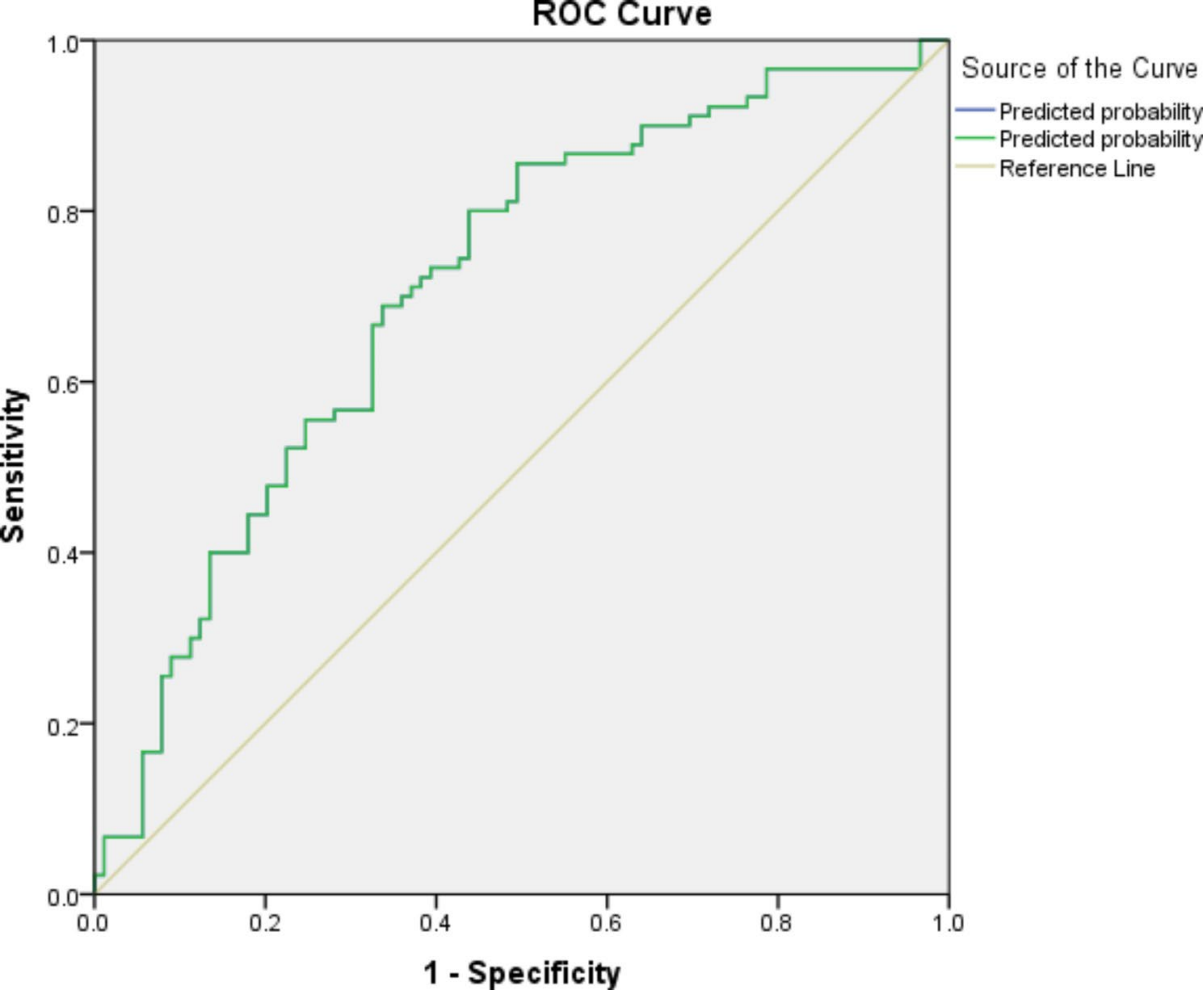
ROC curve to illustrate prediction of free-β-HCG, inhibin and AFP markers with PCOS

Roc curve showed that the prediction power of free-β-HCG, inhibin and AFP markers, which are significant in the second table, is equal to 71%. Considering the cutoff point of 0.5, the sensitivity will be 72% and the specificity will be 62%. In logistic regression, the chance of PCO is the PRIMARY OUTCOME (Fig. 1).

Logistic regression model outcome revealed that the screening parameters of free- β-HCG (OR = 1.020, 95% CI: 1.001–1.040, P = 0.040), inhibin-A (OR = 1.049, 95% CI: 1.016–1.082, P = 0.003) and AFP (OR = 1.32, 95% CI: 1.009–1.055, P = 0.007) were related to PCOS. In addition, one-unit increase in free- β-HCG, inhibin-A and AFP increased the odds ratio of PCOS by 2, 5 and 3% comparing to the control group, respectively. Also OR < 1 had a protective effect, and OR > 1 was a risk factor [16] (Table 3).

**Table 3:** Predictors of the second-trimesters screening of aneuploidy in PCOS and control women using logistic regression model

Variables	Odds Ratio	95% CI	P-value
Free β-HCG (ng/ml)	1.020	1.001–1.040	**0.04**
Inhibin-A (pg/ml)	1.049	1.016–1.082	**0.003**
AFP (ng/ml)	1.032	1.009–1.055	**0.007**

Dependent variable: odds of PCOS: Polycystic Ovary Syndrome,

Independent variables: Free β-HCG: Free Beta-Human Chorionic Gonadotropin, Inhibin-A, AFP: Alpha Fetoprotein, OR: Odds Ratio, CI: Confidence Interval

## Discussion

The current study aimed at assessing the effect of maternal PCOS on the first- and second-trimester screening tests of aneuploidy. The results showed that the mean of free-β-HCG in the PCOS women was higher than in the healthy women, but there was no significant difference in PAPP-A and NT in the first-trimester screening between the two groups. Hacivelioglu et al. [13] reported that the MoM levels of free-β-HCG were higher in the PCOS women than in the control group, and consistent with our results, no difference was found in the MoM levels of PAPP-A and NT between the two groups. In contrary, Karsli et al. [17] showed that the level of free-β-HCG and PAPP-A in women with PCOS was lower than in the control group, and NT measurement result was similar in both groups.

HCG is secreted from the cytotrophoblast cells of placenta during pregnancy [18] and its structure (β-chain) is similar to that of LH [5]. The biological activity of HCG in the first trimester is higher than that of LH [19]. HCG functions via the LH receptor, and The LH activity is mediated by HCG. The predominant form of HCG at the beginning of pregnancy is LH-HCG and both hormones have a membrane receptor [20, 21]. As a result, free β-HCG increased in the first trimester of pregnancy in PCOS women comparing to the control women.

To our knowledge, this is the first study to assess the effect of maternal PCOS on the screening results of biochemical markers in the second trimester. In this study, there was no statistically significant regarding HCG and UE3 in the second trimester between the two groups. However, the mean level of AFP was significantly higher in women with PCOS comparing to the controls. Marin et al. [22] found that vascular injury and fetal hypoxia increased placental inflammation and thrombosis in PCOS women. Additionally, placenta inflammation and thrombosis increased the complications of childbirth, placenta diffusion and uterine-placenta dysfunction in PCOS women. Moreover, delivery complications and uterine-placenta abnormality led to increased AFP levels in the PCOS women comparing to the controls.

There was also a significant association in inhibin-A between the two groups. Inhibin hormone is secreted from antral follicle in the ovary and effects on the pituitary FSH hormone. Placenta inhibin-A hormone is similar to gonad inhibin-A. Inhibin-A decreases hypothalamus Gonadotropin-releasing hormone (GnRH), and FSH increases the ovary granolosa inhibin-A. Also GnRH increases Insulin-like Growth Factor-1 (IGF-1), and IGF-1 in turn increases inhibin-A. Furthermore, women with PCOS have more antral follicles, and thos, more inhibin-B is produced in this women[23, 24]. On the other hand, increased LH-HCG secretion in early pregnancy [4] increases inhibin-Aand decreases inhibin-B. This, in turn, decreases FSH level in women with PCOS [20, 21].

We observed that the risk of trisomy 18 in PCOS women in quad screening was significantly lower than in the controls but there was no significant difference in trisomy 18 risk in sequential screening between the two groups, which is probably false positive, and it is better to dosequential screening in PCOS women, ;however, no association was found between the increased risk of other aneuploidies in the first and secondtrimesters with PCOS. Also no relationship was found between NTD and SLOS risks and PCOS.

In the present study, the risk of pre-eclampsia was higher in the PCOS women than in the controls. Most of PCOS women have metabolic syndrome, insulin resistance, and hypertension. Increased blood pressure can damage the vessels; on the other hand, hypertension and vascular damage are exacerbated in pregnancy. Damage to placental arteries causes the secretion of protein into the mother’s blood-stream and leads to pre-eclampsia [25, 26]. By damaging the placental arteries, inflammatory substances are released and cause placental dysfunction. Placenta injury increases AFP and MPA Levels, leading to the increase of pre-eclampsia. Decreased level of PAPP-A increases the risk of pre-eclampsia, too. The predictive value of these markers is unclear and seems to be more related to placenta dysfunction; however, more research is needed to achieve a clear-cut answer in this regard [15].

As study limitations, we did not investigate the association of the first- and second-trimester screening in PCOS women with different weight and BMI ranges (e.g. obese PCOS and non-obese PCOS); the women’s blood pressure was not measured and pregnancy and neonate outcomes after birth and their relationship with screening were not investigated.

## Conclusions

In conclusion, there was a significant difference in free β-HCG, inhibin-A, AFP level, and the risks of pre-eclampsia and trisomy 18 in PCOS and healthy women; however, no significant difference was observed in other aneuploidies, SLOS risk, NT, PAPP-A, HCG, and UE3 between the two groups. These erorr in PCOS screening may require correction for calculation of risks related to the first- and second-trimester screening for aneuploidy.

## Data Availability

All data generated and analyzed in this study are included in this published manuscript.

## Abbreviations

AFP: Alpha Fetoprotein
β-hCG: Beta-human chorionic gonadotropin
Free β-hCG: Free beta-human chorionic gonadotropin
FSH: Follicle Stimulating Hormone
GnRH: Gonadotropin-releasing hormone
HCG: Human Chorionic Gonadotropin
IGF-1: Insulin-like Growth Factor-1
LH: Luteinizing Hormone
MoM: Multiples of Median
NTD: Neural Tube Defect
PAPP-A: Plasma protein A
PCOS: Polycystic ovary syndrome
SLOS: Smith-Lemli-Opitz Syndrome
UE3: Unconjugated Estriol
